# Age-dependency in mortality of family caregivers: a nationwide register-based study

**DOI:** 10.1007/s40520-020-01728-4

**Published:** 2020-10-11

**Authors:** Tuija M. Mikkola, Hannu Kautiainen, Minna Mänty, Mikaela B. von Bonsdorff, Teppo Kröger, Johan G. Eriksson

**Affiliations:** 1 Folkhälsan Research Center, Helsinki, Finland; 2 Clinicum, Faculty of Medicine, University of Helsinki, Helsinki, Finland; 3 Primary Health Care Unit, Kuopio University Hospital, Kuopio, Finland; 4 City of Vantaa, Vantaa, Finland; 5 Department of Public Health, University of Helsinki, Helsinki, Finland; 6 Gerontology Research Center and Faculty of Sport and Health Sciences, University of Jyväskylä, Jyväskylä, Finland; 7 Department of Social Sciences and Philosophy, University of Jyväskylä, Jyväskylä, Finland; 8 Singapore Institute for Clinical Sciences, Agency for Science, Technology and Research, Singapore, Singapore; 9 Department of Obstetrics and Gynaecology, Yong Loo Lin School of Medicine, National University of Singapore, Singapore, Singapore; 10 Department of General Practice and Primary Health Care, University of Helsinki and Helsinki University Hospital, Helsinki, Finland

**Keywords:** Informal caregiver, Family caregiver, Mortality, Cause of death, Register-based study, Aging

## Abstract

**Background:**

Evidence on family caregivers' health is conflicting.

**Aim:**

To investigate all-cause and cause-specific mortality in Finnish family caregivers providing high-intensity care and to assess whether age modifies the association between family caregiver status and mortality using data from multiple national registers.

**Methods:**

The data include all individuals, who received family caregiver's allowance in Finland in 2012 (*n* = 42,256, mean age 67 years, 71% women) and a control population matched for age, sex, and municipality of residence (*n* = 83,618). Information on dates and causes of death between 2012 and 2017 were obtained from the Finnish Causes of Death Register.

**Results:**

Family caregivers had lower all-cause mortality than the controls over the follow-up (8.1 vs. 11.6%) both among women (socioeconomic status adjusted hazard ratio [HR]: 0.64, 95% CI 0.61–0.68) and men (adjusted HR: 0.73, 95% CI 0.70–0.77). When modelling all-cause mortality as a function of age, younger caregivers had only slightly lower or equal mortality to their controls, but older caregivers had markedly lower mortality than their controls, up to more than 10% lower. Caregivers had a lower mortality rate for all the causes of death studied, namely cardiovascular, cancer, neurological, external, respiratory, gastrointestinal and dementia. The lowest risk was for dementia (subhazard ratio = 0.29, 95% CI 0.25–0.34).

**Conclusions:**

Older family caregivers had lower mortality than the age-matched general population while mortality did not differ according to caregiver status in young adulthood. This age-dependent advantage in mortality is likely to reflect the selection of healthier individuals into the family caregiver role.

## Introduction

Family caregivers, that is those who take care of their relatives or loved ones because of an illness, disability or other specific need for care, form an important source of care in many societies, if not in all societies. A family caregiver can take care of, for example, his/her chronically ill spouse, child with a disability or an older neighbour. As the global population ages the importance of family caregiving is increasing at the societal level. It has been estimated that family caregivers provide the vast majority of long-term care in OECD countries while formal long-term care services only form "the tip of an iceberg" [1, 2]. To promote the continuity of care in the society and the well-being of the family caregivers, it is important to study the health and well-being of family caregivers.

Taking care of another person may bring joy, a feeling of being needed and a sense of purpose in life to the family caregiver [3, 4] but still, several studies report poorer mental health [5, 6], more sleep problems [6], and higher stress levels [5–7] in family caregivers compared to non-caregivers. The findings in relation to physical health outcomes among caregivers compared to non-caregivers are contradictory [8–14]. The majority of these studies have employed subjective physical health indicators and only few have studied risks of specific diseases. Most of the studies investigating disease risks have reported higher risks for cardiovascular diseases in caregivers than non-caregivers [11–14].

In contrast, recent large studies on family caregiver mortality risk have pointed towards lower overall mortality risk in family caregivers compared to non-caregivers [15–17]. Despite this, only little has yet been done to date to explore family caregiver mortality in more detail. There is a paucity in studies investigating causes of death and moderating factors. To the best of our knowledge, only one large study, a census-based record linkage study, has reported mortality risks for different causes of death [15]. However, that study did not report deaths from neurological diseases or dementia, which are prevalent in older age. Investigation of causes of death could give more insight into the mechanisms, which lead to lower mortality in family caregivers compared to non-caregivers. Further, with only a few exceptions [15, 18], most of the previous family caregiver studies have either ignored the potential effect of age on mortality risk or have focused on a narrow age range. Age is a strong predictor of mortality in the general population and hence, its effects should be carefully studied. Exploring age as the moderator of mortality risk might give a deeper understanding on possible subgroups of family caregivers and consequently, give more insight into some of the contradictory findings. Therefore, the purpose of this study was to investigate all-cause and cause-specific mortality in Finnish family caregivers compared to a matched control population over a six-year follow-up using national register-based data. The purpose was also to examine how age modifies the association between family caregiver status and mortality.

## Methods

### Context

This study is based on national Finnish register data. The study aimed at including all individuals in Finland, who were officially recognized family caregivers according to receipt of family caregiver´s allowance in 2012. In Finland, support for family caregiving, including family caregiver's allowance, is regulated by national legislation (Act on Support for Informal Care), which defines the prerequisites for granting support. Family caregiver support is a discretionary social service granted by municipalities (336 municipalities in Finland in 2012) and can be granted for a family caregiver if the care recipient requires demanding care or attendance at home due to functional limitation, illness, disability or other comparable reasons. According to a survey in Finnish municipalities in 2012, a caregiver, applying the definition used in this study, is typically a spouse (58% of the caregivers), parent (23%) or adult child (14%) of the care receiver [19]. The care recipients of the Finnish officially recognized family caregivers typically require care that is of high or moderately high intensity [19]. The minimum level of family caregiver's allowance is defined by legislation, which was 364.30 € per month in 2012 (4372 € per year). Eligibility for family caregiver's allowance is not dependent on the family caregiver's income or employment status.

### Material

The family caregivers were identified from the register of the Finnish Tax Administration. All individuals, who had registered income in the Tax Administration's category "Family caregiver's or private caregiver's allowance" in 2012 were identified. Next, private caregivers could be excluded based on information on receipt of private caregiver's tax deductions because only private caregivers, and not family caregivers, are entitled to these tax deductions. Altogether, 42,372 family caregivers (also 'caregiver' from now on) were identified. Of these caregivers, further register information could not be retrieved for 104 individuals (2 with erroneous personal identity code, 102 had forbidden the disclosure of their personal information for safety reasons). Eight caregivers had died just before January 1, 2012 and were excluded from the study. In addition, four caregivers were excluded because they were classified as being in institutional care (see the criteria later). The final number of caregivers in the study was 42,256 (about 1% of the adult population in Finland).

Two controls—matched according to birth year, sex, and municipality of residence—per one caregiver were drawn from the register of the Population Register Centre. Controls were drawn without replacement and hence, a person could be included in the dataset only once. Caregivers and persons living in the same household than caregiver did not qualify as controls. The index date for control sampling was January 1, 2012. For 28 caregivers, only one matching control subject was found and for 16 caregivers no matching control subjects were found. In total, the data included 84,476 control subjects. We further aimed at removing from the data control subjects, who were in institutional care (*n* = 858) and hence, were not comparable to caregivers. Based on the information obtained from the national Care Register for Social Welfare administered by the National Institute for Health and Welfare, a person was classified as being in institutional care if the person had received a decision on long-term care; or was living in a nursing home; in a 24-h sheltered housing for people with dementia; in service housing for psychiatric patients; or in an institution; in housing for persons with intellectual disability; or if the person was under 65 years and living in a 24-h sheltered housing. The final number of the control population was therefore 83,618.

Data linkages were performed by register-keeping authorities using personal identity codes. Prior to transfer of the data to the investigators, the data were pseudonymised and personal identity codes were removed. The study plan was approved by the Ethics Committee of the Helsinki and Uusimaa Health Care District.

### Date and cause of death

Information on dates and causes of death that occurred between January 1, 2012 and December 31, 2017 were obtained from the Finnish Causes of Death Register, which is maintained by Statistics Finland. The cause of death information in the register is obtained from death certificates. The causes of death are classified according to the International Classification of Diseases (ICD-10). We categorized the causes of death as follows: (1) cardiovascular (ICD-10 codes I00-99), (2) cancer (C00-D48), (3) neurological (G00-99), (4) external (V00-Y98), (5) respiratory (J00-99), (6) gastrointestinal (K00-93), (7) dementia (F01, F02, F03, G30), and (8) other causes of death (the causes not included in other categories).

### Other variables

Information on birth year and date of moving abroad were obtained from the Population Register Centre. Age was calculated as 2012 minus the birth year. Years of education by 2012 were calculated based on the highest degree attained, obtained from Statistics Finland. Information on the annual earned income, caregiver's allowance, and capital income was retrieved from the register of the Finnish Tax Administration. For descriptive purposes, employment status in 2012 was derived from the information obtained from Statistics Finland and income information. The socioeconomic position is determined based on a person's situation in life (economically active, pensioner, etc.), occupation, and occupational status [20]. Socioeconomic position was re-categorised into employment status including three categories (1) employed/student, (2) unemployed/employed part-time, (3) pensioner. A person was classified as unemployed/employed part-time if s/he was unemployed or if the socioeconomic position was unknown and annual earned income was less than 9000 € per year. Those with both unknown socioeconomic position and annual earned income 9000 € or more per year were classified as employed. Information on the degree of urbanisation (urban/semi-urban/rural) of the subjects' municipality of residence was retrieved from the Statistics Finland [21]. The classification is based on the proportion of inhabitants of the municipality living in urban settlements and on the number of inhabitants living in the largest urban settlement of the municipality [22].

### Statistical analysis

Data are presented as means with standard deviation (SD), median with interquartile range (IQR) or as counts with percentages. For the analyses, a new, continuous socioeconomic status (SES) variable was computed based on years of education and income. Creating an average score helped in overcoming the spurious effect that would have resulted from the mutual associations between age, years of education and income; older adults have significantly fewer years of education than younger adults and income drops drastically in older age with retirement. Van der Waerden rank-based normalization was used to yield standardized scores for each of the two variables (years of education and income) [23], and then, the average of these scores was computed. Survival time was calculated as the number of days between 1 January 2012 and death, moving abroad, or the end of the follow-up, 31 December 2017, whichever occurred first. To present cumulative all-cause mortality for the caregivers and controls as a function of follow-up time, Kaplan–Meier curves were created. Cox regression models were used to compute overall hazard ratios (HRs) with accompanying 95% confidence intervals (CIs). Flexible parametric survival modelling (Royston–Parmar models) [24, 25] was used to derive relative difference in mortality between the caregivers and controls and to calculate hazard ratios of all-cause mortality for caregivers (compared to controls) as a function of age at baseline. These models were adjusted for SES. Flexible parametric survival models utilized restricted cubic spline functions in estimation of the associations. Spline functions are piecewise polynomials connected by knots and they allow complex shapes of associations to be modelled. In the present study, four knots were chosen, i.e. there were five piecewise polynomial functions along the baseline age. The knots were located at the 5th, 35th, 65th, and 95th percentiles of baseline age based on Harrell's recommended percentiles [26]. The interaction of caregiver status with the degree of urbanisation of the municipality of residence on mortality was also tested but it was not significant. The proportional hazards assumption was tested graphically and by use of a statistical test based on the distribution of Schoenfeld residuals. Finally, competing risk regression was used to analyse the risk of death due to different causes of death [27]. For each cause of death, the rest of the causes were considered as competing risks. Stata 16.0 (StataCorp LP; College Station, Texas, USA) statistical package was used for the analysis.

## Results

**Fig. 1:**
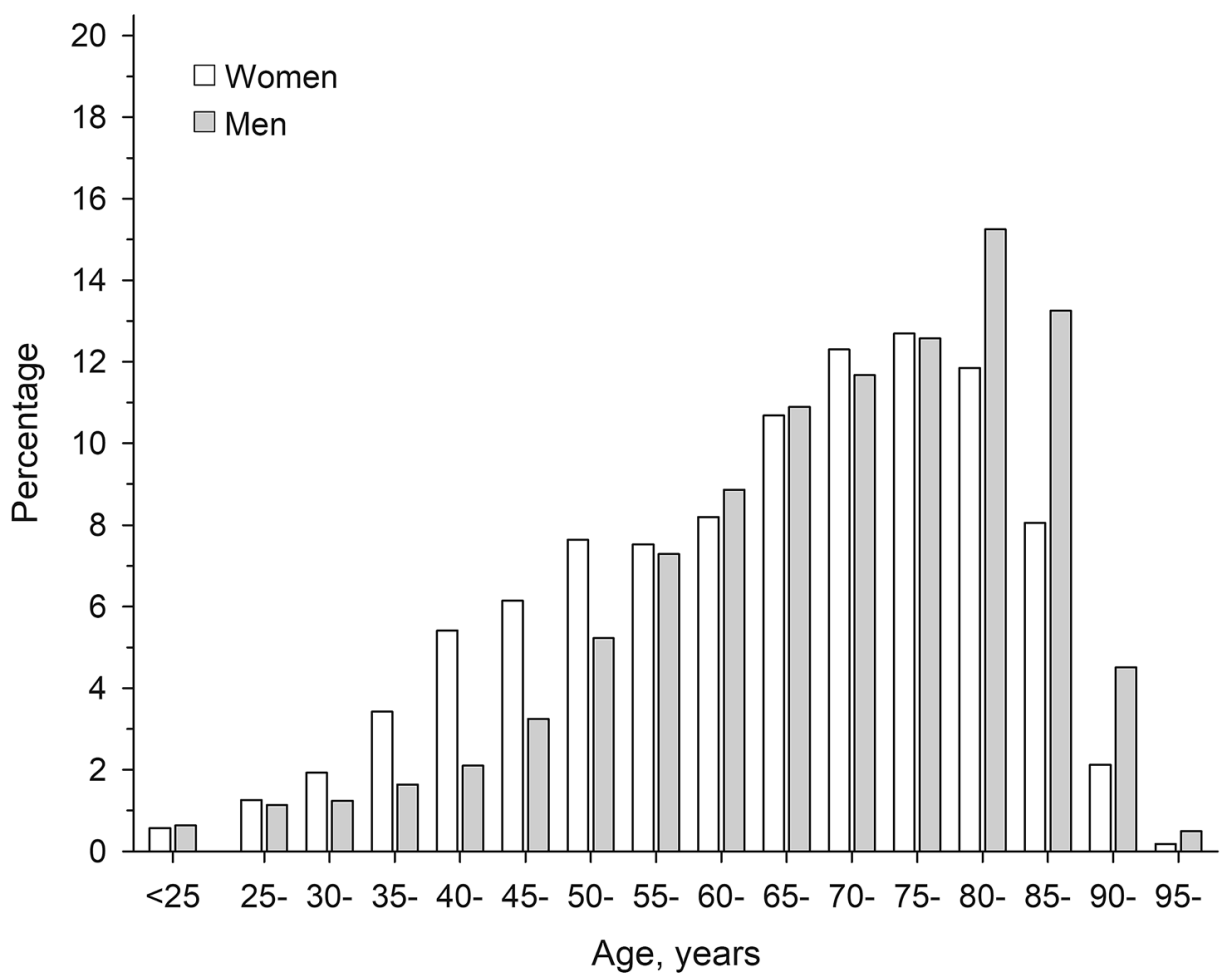
The percentage of female (white bars, *n* = 29,846) and male (grey bars, *n* = 12,410) family caregivers falling into each age category

Figure 1 shows the percentage of the female and male caregivers falling into each age category. Among female caregivers, the most frequent age group was 75–80 years while among the male caregivers, the most frequent age group was 80–85 years. The caregivers had lower educational attainment than the controls (Table 1). Caregivers were less likely to be employed/student and more likely to be retired than the controls. Total annual income in 2012 was similar in caregivers and controls, but when omitting the caregiver's allowance, caregivers had lower median annual income than controls. Median caregiver's allowance in 2012 was equal to the minimum level defined by Finnish legislation.

**Table 1:** Descriptives of family caregivers and the control population at baseline

	Control population *n* = 83,618	Caregivers *n* = 42,256
Women, *n* (%)	59,141 (71)	29,846 (71)
Age, years mean (SD)	67 (16)	67 (16)
Education, mean years (SD)	12.1 (2.8)	11.7 (2.6)
Employment status, *n* (%)		
Employed/student	26,988 (32.3)	11,743 (27.8)
Unemployed/employed part-time	3177 (3.8)	2419 (5.7)
Pensioner	53,453 (63.9)	28,094 (66.5)
Degree of urbanisation in municipality of residence, *n* (%)		
Urban	50,486 (60)	25,485 (60)
Semi-urban	15,139 (18)	7652 (18)
Rural	17,993 (22)	9119 (22)
Income at baseline in euros, median (IQR)		
Total	20,306 (13,168; 31,579)	20,549 (14,975; 29,943)
Without caregiver's allowance	20,306 (13,168; 31,579)	16,075 (10,798; 25,549)

Caregiver's allowance in euros, median (IQR): 4372 (2551, 5400)

**Fig. 2:**
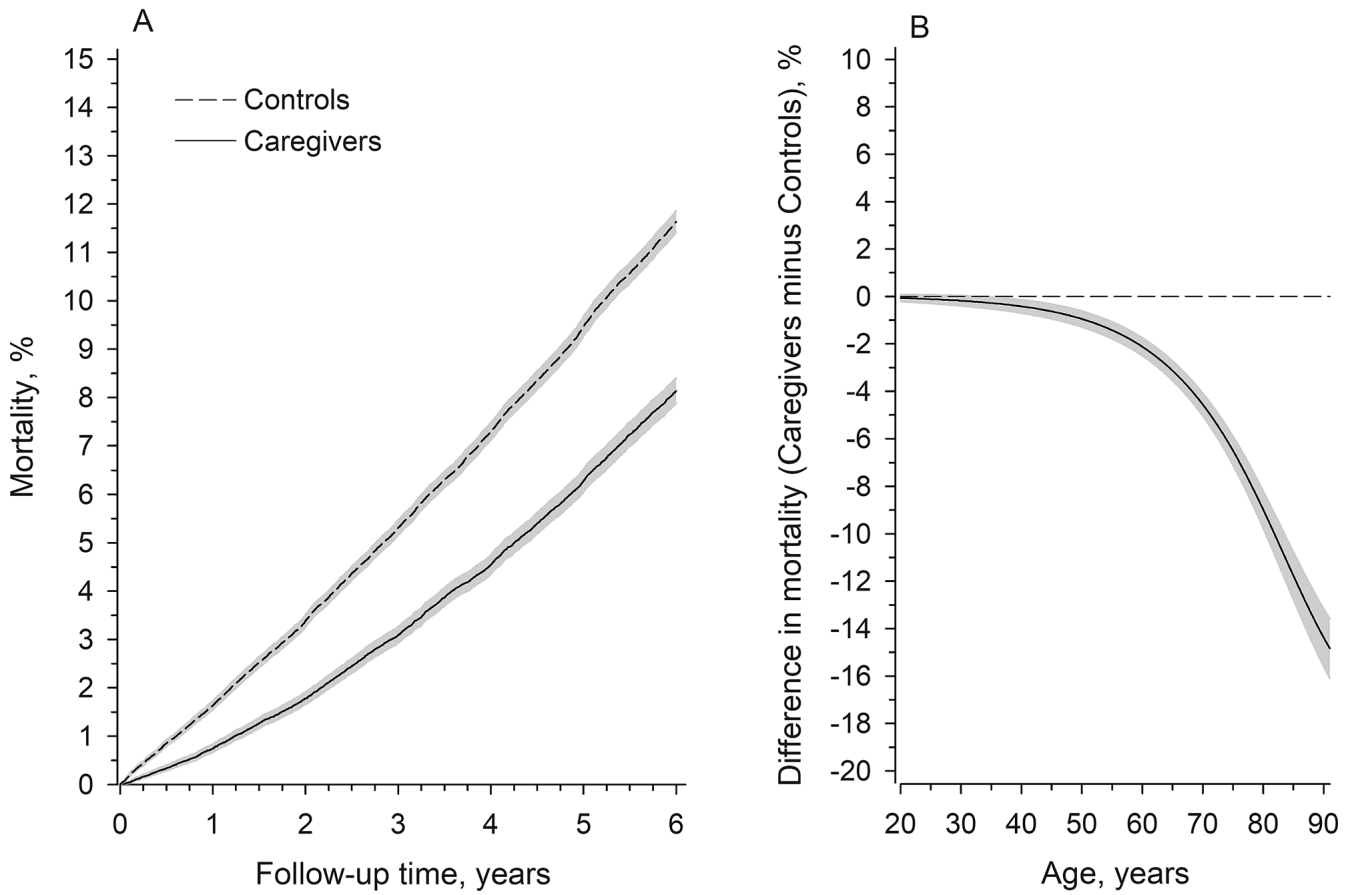
**a** Cumulative all-cause mortality (%) according to follow-up years in caregivers and controls. **b** Relative difference (%) in mortality between the caregivers and controls during the 6-year follow-up according to age at baseline. Shaded areas represent 95% confidence intervals

Caregivers were followed-up for a total of 244,590 person-years (men 69,623 and women 174,967 person-years) and controls were followed-up for a total of 473,287 person-years (men 132,733 and women 340,554 persons-years). Figure 2a presents cumulative all-cause mortality according to follow-up time. Caregivers had lower total mortality over the 6-year follow-up than controls, being 8.1% in the caregivers and 11.6% in the control population. Figure 2b shows the relative difference in mortality between the caregivers and controls during the 6-year follow-up as a function of age at baseline. The figure shows that in younger adulthood there was only a small difference in mortality between the caregivers and controls in favour of the caregivers, if any, but after age 60, the difference increased markedly, resulting in over 10% difference in mortality in the oldest age groups in favour of the caregivers.

**Fig. 3:**
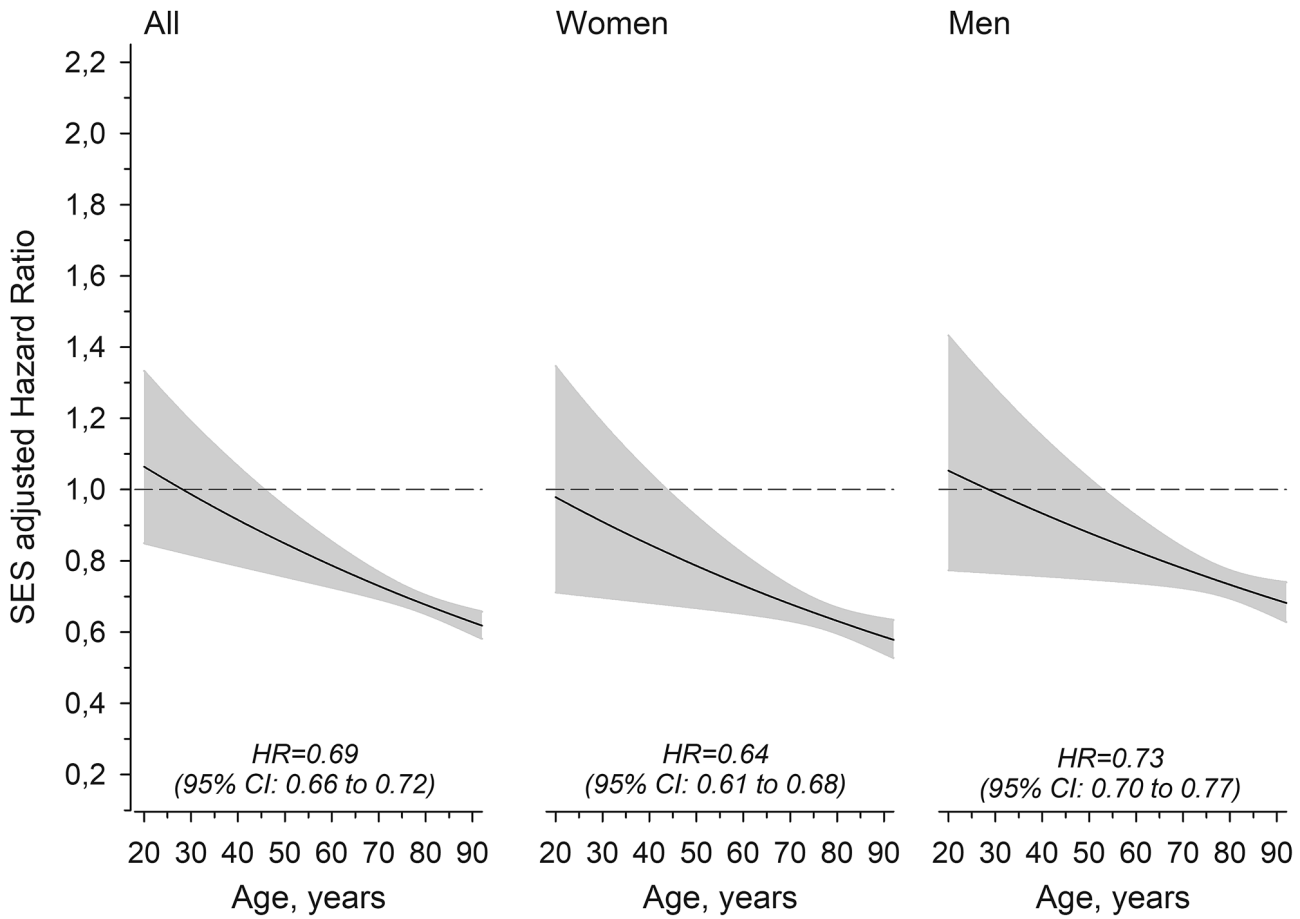
Hazard ratio of all-cause mortality for caregivers compared to controls according to age at baseline. Hazard ratios are adjusted for socioeconomic status (SES). Shaded areas represent 95% confidence intervals

Hazard ratios of all-cause mortality (adjusted for SES) as a function of age at baseline are presented in Fig. 3. The overall hazard ratio was lower for women than for men. However, the figures show that the pattern is similar in women and men: there is no difference in mortality in young adulthood but in older age (after around 50–60 years) caregivers have lower mortality.

Competing risk regression models showed that caregivers have lower risk for all causes of death (Table 2). Adjustment for SES had only negligible effect on the subhazard ratios (sHR), except for cardiovascular mortality. The lowest sHR was found for dementia followed by that of neurological diseases.

**Table 2:** Mortality according to causes of death

	Control population	Caregivers	sHR (95% CI)^b^	
	*n* (%)^a^	*n* (%)^a^	Crude	Adjusted for SES
All deaths	9714	3434		
Cardiovascular	3864 (39.8)	1430 (41.6)	0.73 (0.68–0.77)	0.85 (0.71–0.90)
Cancer	2683 (27.6)	1163 (33.9)	0.85 (0.80–0.91)	0.86 (0.81–0.93)
Neurological	294 (3.0)	73 (2.1)	0.49 (0.38–0.63)	0.49 (0.38–0.64)
External	449 (4.6)	170 (4.9)	0.75 (0.63–0.89)	0.76 (0.64–0.91)
Respiratory	426 (3.4)	152 (4.4)	0.70 (0.59–0.85)	0.74 (0.61–0.89)
Gastrointestinal	336 (3.5)	146 (4.2)	0.86 (0.71–1.04)	0.89 (0.73–1.08)
Dementia^c^	1282 (13.2)	182 (5.3)	0.28 (0.24–0.33)	0.29 (0.25–0.34)

^a^Percentage of the total number of deaths within the group (control population/caregivers)

^b^Subhazard ratio, competing-risks regression model was used where the rest of the causes of death were considered as competing risks

^c^Includes ICD-10 codes F01, F02, F03, and G30 (note: G30 is also included in Neurological category)

## Discussion

We aimed at investigating mortality of Finnish caregivers using national register-based data covering all officially recognized family caregivers in Finland. The results showed that all-cause mortality of the caregivers was lower compared to an age- and sex-matched control population. However, mortality risk of caregivers varied across the age range. Younger family caregivers had only slightly lower or equal mortality than their controls, but older family caregivers had clearly lower mortality than their controls. In addition, family caregivers' risk of death was lower for all causes of death and it was particularly low for dementia and neurological diseases.

Several large population-based studies have reported lower all-cause mortality rates in caregivers than non-caregivers [15–18, 28] while a few smaller studies have found no differences between caregivers and non-caregivers [29–31]. Pooled mortality estimates in an earlier review [32] and a in a recent meta-analysis [33] also indicated that caregivers have a mortality advantage, which is in line with our findings. In the present study, the overall mortality risk of caregivers was only slightly lower (HR = 0.69) than in previous large studies, which have adjusted for self-reported health status at the start of the follow-up [15, 17]. In a study based on census data from England and Wales, mortality of caregivers varied between 0.74 and 0.87 [17], while in the study based on Northern Ireland Census 2011 it was 0.72 [15].

Mortality risk of caregivers was not constant across the age groups. In younger adults, mortality was similar to that of the control population. The difference in mortality between the caregivers and control population started to emerge after the age of 40 years in favour of the caregivers, and the difference widened rapidly after the age of 55–60 years resulting in markedly lower mortality in caregivers compared to the control population, being more than 10% lower in the oldest age groups. The Northern Ireland Census 2011 study reported a higher mortality risk in caregivers compared to non-caregivers among participants younger than 25 years, no difference in participants aged 25–44 years, and a lower mortality risk in caregivers aged 45 years and older [18]. Hence, the pattern that the advantage in mortality in caregivers starts to emerge around 40 to 50 years of age was similar to the findings in our study. This was despite the fact that the age distributions were different in these studies. In the Northern Ireland Census studies, the proportion of persons 65 years and older among the caregivers was 12% in 2011 and 17% in 2001, while the proportion in the present study was 61%. As indicated by the wider confidence intervals, the statistical power of our analysis was lower in the youngest age groups because of the low number young officially recognized caregivers and the low mortality in this age group. On the other hand we were able to analyse mortality risk up to very old age groups.

We argue that the age-dependency of mortality differences according to caregiver status observed in the present study is an indication of "healthy worker effect" or in this case "healthy caregiver effect". "Healthy worker effect" originates from occupational epidemiology and means that healthier individuals are more likely to become employed and stay at work than the less healthy [34]. Analogously, individuals with better health and functioning are more likely and able to take on care responsibilities than their less healthy peers, which would lead to an association observed between being a caregiver and better health. This effect would be seen as improving health or declining mortality relative to the general population with increasing age because morbidity and mortality of the general population increases markedly as a function of age. In younger adults, morbidity and disability in the general population is low and hence, almost anyone can qualify as a caregiver because nearly all individuals are in sufficiently good health. This results in similar mortality in caregivers and non-caregivers in young adulthood. In older adulthood, morbidity, disability, and mortality increase markedly in the general population and therefore, caregivers must be among the healthiest individuals in their age group in order to manage care responsibilities. The existence of the "healthy caregiver effect" was also supported by findings of a previous study, in which physically healthier older individuals were more likely to become family caregivers and stay in that role than those with poorer physical health [35]. The authors concluded that the "healthy caregiver effect" may substantially underestimate the negative impact of caregiving on health. It is also possible that older caregivers get a sense of purpose in life from caregiving, which they may miss after retirement. The sense of purpose may have a positive impact on those caregivers' health, as discussed previously in several studies [4, 15, 16]. However, the majority (72%) of the working aged caregivers (that is under 65 years), were employed or students. These working caregivers may suffer from stress arising from negotiating competing demands of caregiving and work [36], and may therefore be more negatively affected by caregiving than older, retired caregivers.

Cause-specific mortality of caregivers showed marked variation. We are aware of only one previous study with a large enough sample size, which has investigated different causes of death in family caregivers [15]. The hazard ratios in the present study for cardiovascular, respiratory, gastrointestinal diseases, cancer and external causes were consistent ranging from 0.74 to 0.89. This is well in agreement with the previous study, which reported hazard ratios between 0.71 and 0.86 for cardiovascular diseases, respiratory diseases and cancer in a subgroup of caregivers with the most time consuming care responsibilities in comparison to non-caregivers [15]. Leggett and others studied spousal dementia caregivers and reported subhazard ratios ranging between 0.69 and 0.70 for cancer, cardiovascular, and cerebrovascular diseases as compared to non-caregivers [37], while a Japanese study reported a higher likelihood of CVD mortality in caregivers than in non-caregivers, but only among women and no differences in cancer mortality [29]. The estimates from these two studies had, however, wide confidence intervals signifying high uncertainty. In the present study, hazard ratios for neurological diseases (sHR = 0.49) and dementia (sHR = 0.29) deviated substantially from those for the other causes of death. Mortality risk for neurological diseases and dementia have not been reported, to our knowledge, in previous large studies on caregivers although Alzheimer's disease and other dementias are the fourth leading cause of death in the high-income countries [38]. The low mortality from dementia and neurological diseases in caregivers also support the "healthy caregiver effect" as an explanation for the results because a person with advanced dementia is very unlikely to become a caregiver. Some of the previous studies have aimed at adjusting for baseline health using self-reports on limiting long-term illness and perceived general health. However, this is unlikely to remove the "healthy caregiver effect" because first, the assessment of health status has not been done before the selection to caregiving, and second, self-reports are unlikely to account for all the differences in disease type and severity between the caregivers and non-caregivers. Previous studies have yielded small or negligible changes in hazard ratios (change in HR 0.02–0.13) after adjustment for self-reported health [15, 17].

Health effects of caregiving may depend, among other factors, on the level of commitment required by the care and this may contribute to the differences in published studies. The criteria for granting caregiver's allowance in Finnish municipalities are strict, and they usually require everyday involvement in care responsibilities. This strictness of the criteria is reflected by the relatively small number of caregivers receiving the caregiver's allowance, about 1% of the adult population. Previous studies have, in turn, defined caregiving in a way that it also includes very low intensity caregiving [15–17, 28, 29], for example one hour per week. It appears that when caregiving has been defined in this broad manner the majority of the caregivers report providing relatively little care. For example, in Ireland Census 2011 the vast majority of caregivers provided care 1–19 h *per week* [15] while a survey in Finnish officially recognized caregivers in 2012 reported that 69% of caregivers provided care 13–24 h *per day* [39]. According to the same survey, 90% of the Finnish officially recognized caregivers lived in the same household as the care recipient, and the care recipient was most frequently the caregiver's spouse and second most frequently the caregiver's child, which may be more demanding than other family care relationships [40]. In a study by Roth and colleagues [28], over 30% of the caregivers were providing care to their parents and 22% to their spouses. Large surveys using broad definitions of caregiving report that typically a care recipient is a parent or parent-in-law [29, 41, 42] and that caregivers do not typically live with their care recipients [42]. Hence, our study differs from most previous studies and can be considered a study focusing on 'high-intensity' caregivers. When comparing hazard ratios of the present study to the group considered to have the heaviest care responsibilities (50+ h per week) in the Northern Ireland Census 2011 (HR = 0.76), we can conclude that the estimates are comparable [15].

The strengths of this study include the large sample comprising all officially recognized family caregivers in Finland. We had reliable information on the causes of death and we also investigated deaths due to dementia and neurological diseases, not studied previously in relation to family caregiving. Our control population was matched for key demographic characteristics. There are also some limitations. Because most of the care recipients of the official Finnish family caregivers require care that is of high or moderately high intensity [19] the results of the present study are only applicable to family caregivers, who have demanding and time-consuming care responsibilities on a daily basis. Although we included all officially recognized family caregivers into this analysis, there may be family caregivers who carry out demanding and binding care and therefore, could qualify as official caregivers, but have not got the status of an officially recognised caregiver. Carers Finland, an advocacy and support association for family caregivers, has estimated that there are about 16,000 such family caregivers in Finland. These caregivers could be in poorer health than caregivers receiving caregiver's allowance as they may be out of reach of other support services also. In this case, the results would overestimate survival of the family caregivers. When interpreting the findings it should be noted factors such as societal context, culture and availability of formal care influence the volume of family care provided in each country, which, in turn, may influence health of the caregivers [43]. Finland can be described as a service-based welfare state, where formal services are emphasized and obligation to care for a disabled relative is low [44].

## Conclusions

Older family caregivers have markedly lower mortality than the age-matched general population but younger family caregivers have equal or only slightly lower mortality than an age-matched population. Age-dependency in relative mortality risk is likely to reflect the selection of healthier individuals into the family caregiver role. Possibility of this selection should be borne in mind when interpreting results of studies focusing on health of family caregivers.

## Data Availability

The data that support the findings of this study are available from Finnish Tax Administration, and Findata but restrictions apply to the availability of these data, which were used under license for the current study, and so are not publicly available. Data are however available from the authors upon reasonable request and with permission of Finnish Tax Administration and Findata.
